# The Incidence and Prognostic Value of Hypochloremia in Critically Ill Patients

**DOI:** 10.1100/2012/474185

**Published:** 2012-06-04

**Authors:** Makiko Tani, Hiroshi Morimatsu, Fumiaki Takatsu, Kiyoshi Morita

**Affiliations:** ^1^Department of Anesthesiology and Resuscitology, Okayama University Hospital, 2-5-1 Shikata-cho, Kita-ku, Okayama 700-8558, Japan; ^2^Perioperative Management Center, Okayama University Hospital, 2-5-1 Shikata-cho, Kita-ku, Okayama, Japan; ^3^Medical School, Okayama University, 2-5-1 Shikata-cho, Kita-ku, Okayama, Japan

## Abstract

Little is known on the clinical effects of chloride on critically ill patients. We conducted this retrospective, observational study in 488 critically ill patients to investigate the incidence of chloride abnormalities, effects of hypochloremia in acid-base disorders, and association between chloride and clinical outcome. The study involved retrieval of arterial blood gas analyses, biochemical and demographical data from electrical records as well as quantitative acid-base analyses. For statistical analysis, the patients were stratified into three groups according to their chloride level (normal range: 98–106 mEq/L). The distribution of chloride levels was hyperchloremia 16.6%, normochloremia 74.6%, and hypochloremia 8.8%. The hypochloremic group was significantly alkalemic (*P* < 0.0001) and has significantly higher apparent strong ion difference (SIDa) (*P* < 0.0001) compared to the two other groups. The hypochloremic group had significantly longer stays in the ICU and hospital (*P* < 0.0001) with higher mortality (*P* < 0.0001). However, multiple regression analysis showed that chloride was not an independent factor of poorer outcome. In conclusion, the acid-base characteristics of the hypochloremic patients were alkalemia coexisting with higher SIDa. And although it was not an independent prognostic factor, hypochloremia was related to poorer outcome in critically ill settings.

## 1. Introduction

Abnormalities in acid-base and electrolytes are common in critical care settings, and it is therefore important that these abnormalities are understood and interpreted correctly to ensure appropriate treatment of patients. Stewart's physicochemical approach [1, 2] is considered to provide more specific analysis and insights into the pathogenesis of acid-base disorders [3]. Stewart suggested that PaCO_2_, a strong ion difference (SID) and total weak acid concentration were the three independent variables that controlled hydrogen ion and bicarbonate concentrations [2].

Disparity of positive and negative charges of strong ions is termed SID with chloride and sodium being the two main elements that determine SID [1, 2]. This is because chloride is a strong anion that provides a large amount of negative charge in extracellular fluid [4]. As a consequence, chloride plays an important role in acid-base balance. However, chloride has received less attention than other ions, and acid-base analysis is often not carried out from the perspective of chloride levels. As a result, many questions remain to be answered, such as the incidence of chloride abnormalities and effects of these changes on acid-base balance and clinical outcome in critically ill patients [5].

This study aimed to assess the significance of chloride levels in critically ill patients by determining the incidence of chloride abnormalities, characteristics of acid-base balance, and clinical outcome in critically ill patients.

## 2. Materials and Methods

The study was approved by the Institutional Review Board of Okayama University Hospital in Japan. Because all data used in the study were collected during routine practice, informed consent was waived.

### 2.1. Study Protocol

We carried out a retrospective examination of critically ill patients in a 22-adult bed medical and surgical intensive care unit (ICU) at Okayama University Hospital. Standard patient care in all patients included collecting arterial blood samples for arterial blood gas and biochemical analyses on admission and daily thereafter. All arterial blood gas analysis data were stored electronically and available on our computer with the patients' ID, measurement date and time.

We extracted data of patients who were in the ICU from January to December 2009. This data included arterial blood gas analysis (pH, PaCO_2_, PaO_2_, and bicarbonate) with simultaneous standard base excess (SBE), anion gap (AG), hematocrit, sodium, potassium, chloride, ionized calcium, lactate, and glucose levels in arterial blood. We also obtained the blood biochemistry data of each patient from ICU records, including albumin, phosphate, and total bilirubin. Demographic data was also obtained from the records, including age, sex, admission diagnosis, and Acute Physiology and Chronic Health Evaluation (APACHE) II score on ICU admission.

The data list included several series of data for one patient. The series of results with the lowest chloride level was used in the analyses.

### 2.2. Study Population

Patients admitted to the ICU between January and December 2009 were considered eligible for the study if they were older than 16 years and had stayed in the unit for longer than 24 h, with arterial blood gas and biochemistry having been checked at least once. If patients did not fulfill the inclusion criteria as above, they were excluded from the analysis.

### 2.3. Laboratory Techniques and Measurements

Arterial blood gas was analysed, and sodium, potassium, chloride, calcium, lactate, and glucose levels were measured using an ABL800 (Radiometer, Copenhagen, Denmark). Albumin, phosphate, and total bilirubin were measured in the central laboratory of our hospital using a JCA-BM2250 BioMajesty (Nihondenshi, Tokyo, Japan).

### 2.4. Quantitative Acid-Base Analysis

The quantitative physicochemical approach of Stewart, modified by Figge et al. [6–8] was used to assess acid-base disorders. This method is characterized by consideration of the effects of plasma proteins and plasma weak acids (PaCO_2_, albumin, and phosphate). Standard formulas used in this study were as follows:

Apparent strong ion difference (SIDa) = SID = [Na^+^]+[K^+^]+[Ca^++^]−[Cl^−^]−[lactate] (all concentrations in mEq/L).

Effective strong ion difference (SIDe) = 2.46 × 10^−8^ × PaCO_2_/10^−pH^ + [albumin] × (0.123 × pH–0.631) + [phosphate] × (0.309 ×pH–0.469)  (PaCO_2_ in mmHg, albumin in g/L, phosphate in mmol/L).

The strong ion gap (SIG) = SIDa − SIDe.

### 2.5. Statistical Analysis

The data are presented as mean ± standard deviation. For statistical comparison, the patients were divided into three groups according to their lowest chloride level: hypochloremia (<98 mmol/L), normochloremia (98–106 mmol/L), or hyperchloremia (>106 mmol/L). We set the normal range according to the values listed by the manufacturer of the blood gas analyzer set. The primary outcomes were length of stay and mortality in the ICU and hospital. We also analysed the characteristics of the acid-base balance in each group. Analysis of variance (ANOVA), and Tukey's-HSD test were used to compare the acid-base variables and length of stay in the ICU and hospital in the three groups. Chi-square test was used to assess mortality in the ICU and hospital, while multiple logistic regression models were used to determine the independent predictors of survival. Pearson's correlation coefficients were calculated to examine the relationship between APACHE II score and chloride levels. The statistical analyses were performed using JMP6 (SAS Inc, Cary, NC, USA), and *P* < 0.05 was considered statistically significant.

## 3. Results

**Figure 1:**
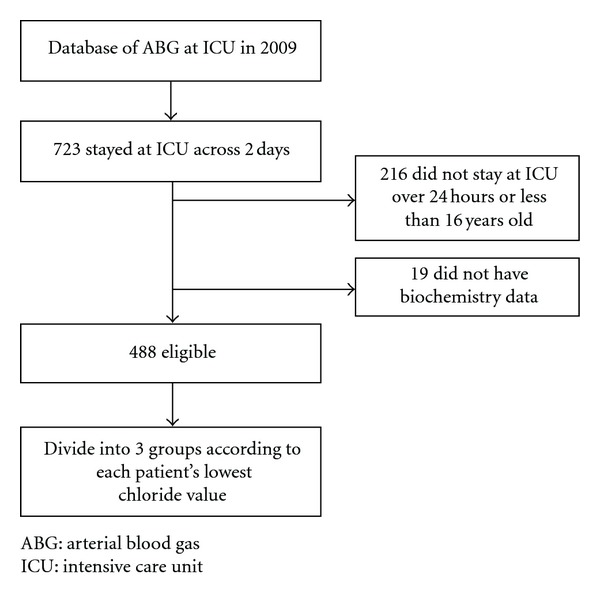
Flow diagram of patients selection during the study.

723 patients stayed at the ICU across two days. Nineteen patients were excluded because of lack of biochemistry data and 216 due to their age or short ICU stay. A total of 488 patients were eligible for the study and were included in the final analysis (Figure 1). Their general characteristics and clinical variables are shown in Table 1. Eighty-one patients (16.6%) were classified as having hyperchloremia, 364 patients (74.6%) with normochloremia, and 43 patients (8.8%) with hypochloremia.  Table 2 shows the acid-base characteristics of the three groups. The acid-base variables in hypochloremic patients were significantly different from the hyperchloremic and normochloremic patients. Hypochloremic patients were more alkalemic than the hyperchloremic and normochloremic patients. Over half of the hypochloremic patients with alkalemia had a high SBE. Using Stewart's approach, a high SIDa (>37 mEq/L) was found in 24 patients (55.8%). Sodium levels were also lower in about 50% of the hypochloremic group. However, the sum of strong anions was low in this group, indicating that the cause of the high SIDa may be a low chloride concentration.

Table 3 shows a comparison of the length of stay and mortality in the ICU or hospital according to chloride levels. Hypochloremic patients stayed significantly longer in both the ICU and hospital, with their mortality in both areas being significantly higher than in the other two groups. These results suggest that hypochloremia was related to poor outcome in critically ill patients.

To confirm this relationship between hypochloremia and poor outcome, we performed the following analyses: a comparison between survivors and nonsurvivors in the hospital showed significant differences in APACHE II score, type of admission, PaCO_2_, sodium, potassium, calcium, chloride, bicarbonate, lactate, SBE, AG, and hematocrit (Table 4). The multiple regression model excluding confounding factors, such as SBE and AG confirmed that the APACHE II score, type of admission and lactate were associated independently with mortality. However, chloride level showed no such association with mortality (Table 5).

**Figure 2:**
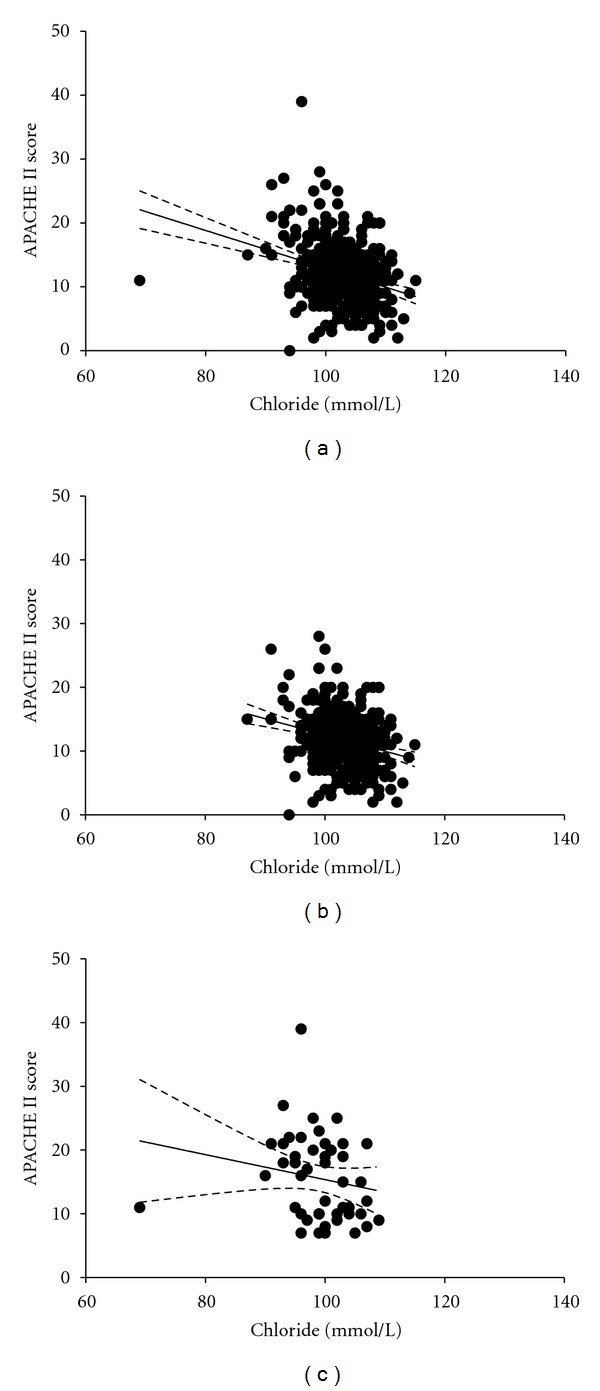
Relationship between chloride and APACHE II score. The solid line represents the linear regression line, and the dotted line represents the 95% confidence band. (a) Total study population. (b) Surgical patients. (c) Medical patients.

We also compared the characteristics of medical or surgical patients. This showed that chloride level was significantly lower in medical admissions (Table 6). This finding suggested that the relationship between chloride levels and severity of patients' condition may differ according to the type of admission. We therefore also examined the relationship between chloride and APACHE II score according to type of admission. Chloride level showed significant correlations with APACHE II score in the study population (*r* ^2^ = 0.085, *P* < 0.0001, Figure 2(a)). This result showed that chloride level was associated with the severity of the medical condition. Specifically, the severity of the conditions was greater in hypochloremic patients in a critical care setting. We also analyzed the data according to admission type and showed there was a significant correlation between chloride levels and APACHE II score in surgical patients (*r* ^2^ = 0.063, *P* < 0.0001, Figure 2(b)). However, there was no correlation between chloride levels and APACHE II score in medical patients (*r* ^2^ = 0.034, *P* = 0.2163, Figure 2(c)). In summary, only surgical patients showed a significant negative correlation between chloride levels and APACHE II score, whereas medical patients tended to have lower chloride levels.

## 4. Discussion

Chloride abnormalities in critical care settings have received considerable attention, especially hyperchloremia as a cause of metabolic acidosis [9] and hypochloremia as a cause of metabolic alkalosis [10–15]. However, chloride abnormalities themselves have not been studied sufficiently. This retrospective, observational study was carried out on critically ill adult patients in a tertiary intensive care unit. The purpose of the study was to understand the nature of chloride abnormalities in critically ill patients, particularly from the viewpoint of hypochloremia. We showed that the prevalence of chloride abnormalities in the critical care setting was 25.4% (hypochloremia 8.8% and hyperchloremia 16.6%). Hypochloremic patients had metabolic alkalemia, and the nature of their acid-base disorders was distinct from those in nonhypochloremic patients. Hypochloremic patients tended to have a poorer outcome.

We are unaware of any other study on the prevalence of hyperchloremia or hypochloremia in the critical care setting, although there is one study on the incidence of hyperchloremia in the intraoperative setting [16]. This study showed that 31.7% of the study population was hyperchloremic, a prevalence approximately 1.9 times greater than the rate observed in our study. However, they administered normal saline as intraoperative fluid, whereas we administered acetate ringer solution mainly as volume therapy. It is possible that this is the reason for the large difference in the incidence of hyperchloremia between the two studies. However, the earlier study set the cut-off value between hyperchloremia and normochloremia at 114 mEq/L, which represented the point where hyperchloremia had the greatest sensitivity and specificity for death. Considering that their mean chloride level was 112.0 ± 6.7 mEq/L compared to 102.7 ± 4.4 mEq/L in our study, it appears that we were studying different populations. It is also possible that different fluid therapies may cause variability in the prevalence of chloride abnormalities.

We found that hypochloremic patients were more alkalemic and had a particularly distinct acid-base status compared to normochloremic and hyperchloremic patients. According to the quantitative physicochemical approach of acid-base balance [2], [H^+^] decreases when SIDa increases, while [H^+^] increases when SIDa decreases. Chloride levels are an important determinant of SIDa. In general, hyperchloremia induces metabolic acidosis by decreasing SIDa, whereas hypochloremia induces metabolic alkalosis by increasing SIDa. These principles were followed in our study. SIDa was significantly higher in the hypochloremic group compared to the other two groups, with a mean value of 4.98 mEq/L. The contributing effects to SIDa were calculated by subtracting the mean value of the nonhypochloremic group from that of the hypochloremic group (−3.14 mEq/L for sodium, 9.56 mEq/L for chloride and −0.95 mEq/L for lactate). Our quantitative analysis using Stewart's approach therefore demonstrated that alkalemia in hypochloremic patients was due mainly to hypochloremia.

We also investigated the relationship between chloride levels and outcomes. Recently, hyperchloremic acidosis was reported to be associated with poor outcomes. Boniatti et al. found that hyperchloremia was associated with mortality in critically ill patients [17], while an experimental study in a rat model of severe sepsis showed that severe hyperchloremic acidemia induced by HCL infusion caused hypotension [18]. In contrast, an observational study on major vascular trauma patients reported no correlation between hyperchloremia and mortality [19]. The relationship between hyperchloremia and outcome therefore remains controversial.

Our data showed that the hyperchloremic group tended to stay in the ICU and hospital for a shorter period of time than the normochloremic group. The ICU and hospital mortality of hyperchloremic patients was almost similar to those of normochloremic patients (Table 3). Although there is no obvious reason why our hyperchloremic subjects did not have poorer outcomes, it is possible that the low cut-off level for hyperchloremia may have contributed to this result. On the other hand, hypochloremic patients showed a tendency for longer ICU and hospital stays and higher mortality (Table 3). Although hypochloremic patients showed metabolic alkalosis, it was difficult to ascertain which is the main factor for the poorer outcome, hypochloremia, or metabolic alkalosis? If we divided patients by metabolic acid-base status using base excess, patients with metabolic acidosis showed the highest mortality (metabolic acidosis 17.4%, metabolic alkalosis 3.9%, normal 6.27%, and *P* value = 0.0265) in the overall patients. Even if we further analyse this trend in each chloride subgroup (hyperchloremia, normochloremia, and hypochloremia), patients with metabolic acidosis showed higher mortality in all subgroups. These results suggest that hypochloremia might be associated with the poorer outcome independently of their metabolic alkalemia.

However, our multivariate analysis showed that hypochloremia itself was not an independent predictor for mortality. As mentioned above, we also demonstrated a different relationship between chloride levels and APACHE II scores according to the type of admission. In summary, hypochloremia may be an important clinical factor for predicting increased mortality risk. Further investigations are therefore needed to clarify the significance of chloride in both surgical and medical patients.

There were several limitations in this study. First, this was a retrospective-observational study susceptible to selection and information bias. However, we selected patients according to predefined criteria, with only a small number of patients (19, 2.6%) being excluded because of loss of biochemistry data, which would not have affected our findings. Second, the strategies used for fluid resuscitation were not standardized. Acid-base variables are affected by the solutions administered [20], and the type of solution used for resuscitation is also important. We did not administer saline for resuscitation, but mainly used acetate-ringer solution and partially used colloids, such as 5% albumin or 6% hydroxyethyl starch (70/0.5/4). Although it is unclear whether and how the type of solution may affect acid-base variables, it is important to note that the incidence of chloride abnormalities observed in our study occurred with administration of acetate-ringer rather than saline solution. Third, we did not carry out sequential measurement of the acid-base variables and only used data associated with the lowest chloride level in each patient. Sequential measurement and average chloride levels may have provided more detailed results; however, it is cumbersome and not practical to analyze previous chloride values and calculate mean chloride values in clinical practice. The lowest chloride value in each patient may be a more simpler and convenient index to predict outcome in clinical settings. Given the limitations of our study, a further prospective clinical trial using a standard fluid protocol is required to obtain definitive findings.

## 5. Conclusions

This observational study on 488 critically ill patients admitted to the ICU showed that hypochloremia is associated with poorer outcomes. Despite the fact that hypochloremia was not an independent factor for higher hospital mortality, hypochloremic patients were more prevalent in medical admissions, with hypochloremia correlating significantly with higher APACHE II scores. This study indicates that hypochloremia has clinical importance as an indicator of prognosis in critically ill patients.

## Figures and Tables

**Table 1:** Patient demographics and clinical variables.

Characteristics	*P *value
*n*	488
Age, years,	61.8 ± 16.2
Sex, male, *n *(%)	293 (60.0)
APACHE II score	12.1 ± 4.4
Reason for ICU admission	
Postoperative, *n *(%)	443 (90.8)
Sepsis, *n *(%)	16 (3.3)
Respiratory failure, *n *(%)	9 (1.8)
Cardiogenic shock, *n *(%)	8 (1.6)
Hypovolemic shock, *n *(%)	3 (0.6)
Miscellaneous, *n *(%)	25 (5.1)
Type of admission, *n *(%)	
Surgical	443 (90.8)
Medical	45 (9.2)
ICU stay, days	7.4 ± 9.6
Hospital stay, days	41.8 ± 39.7
ICU mortality, *n *(%)	15 (3.0)
Hospital mortality, *n *(%)	28 (5.7)

APACHE II score: acute Physiology and Chronic Health Evaluation II score; ICU: intensive care unit.

**Table 2:** Acid-base variables in hyperchloremic, normochloremic, and hypochloremic patients.

Variables	Hyperchloremia (*n* = 81)	Normochloremia (*n* = 364)	Hypochloremia (*n* = 43)	*P* value
pH	7.41 ± 0.04	7.42 ± 0.035	7.44 ± 0.065	<.0001
PaCO_2_, mmHg	38.0 ± 4.8	40.7 ± 5.0	42.3 ± 7.3	<.0001
Bicarbonate, mmol/L	23.8 ± 2.2	26.0 ± 2.4	28.5 ± 5.0	<.0001
AG, mmol/L	7.0 ± 3.3	7.9 ± 2.9	11.3 ± 6.9	<.0001
SBE, mmol/L	−0.16 ± 2.1	2.0 ± 2.3	4.4 ± 5.1	<.0001
Sodium, mmol/L	135.7 ± 3.1	132.6 ± 3.4	130.0 ± 7.0	<.0001
SIDa, mmol/L	30.5 ± 3.1	33.9 ± 3.5	38.2 ± 4.4	<.0001
SIDe, mmol/L	26.9 ± 0.3	29.0 ± 0.2	32.1 ± 0.5	<.0001
SIG, mol/L	3.5 ± 2.8	4.8 ± 2.7	6.2 ± 3.4	<.0001
Albumin, g/dL	2.97 ± 0.57	2.78 ± 0.47	3.0 ± 0.08	<.0001
Phosphate, mmol/L	1.1 ± 0.3	1.00 ± 0.3	1.2 ± 0.5	<.0001
Lactate, mmol/L	1.4 ± 0.99	1.2 ± 0.8	2.2 ± 4.4	<.0001
Lactate + Chloride,	110.1 ± 2.1	103.6 ± 2.6	96.1 ± 6.3	<.0001
mmol/L				

AG: anion gap; SBE: standard base excess; SIDa: apparent strong ion difference; SIDe: effective strong ion difference; SIG: strong ion gap.

**Table 3:** Length of stay and mortality in hyperchloremic, normochloremic, and hypochloremic patients.

Variables	Hyperchloremia (*n* = 81)	Normochloremia (*n* = 364)	Hypochloremia (*n* = 43)	*P *value
ICU stay, days	4.4 ± 2.5	7.3 ± 9.6	14.3 ± 13.3	<.0001
Hospital stay, days	28.4 ± 19.5	41.4 ± 37.3	70.5 ± 65.7	<.0001
ICU mortality, %	2/81 = 2.5	7/364 = 1.9	6/43 = 14.0	<.0001
Hospital mortality, %	3/81 = 3.7	14/364 = 3.8	10/43 = 23.3	<.0001

ICU: intensive care unit.

**Table 4:** Comparison of variables in surviving and nonsurviving patients in the hospital.

Variables	Nonsurvivors (*n* = 28)	Survivors (*n* = 460)	*P* value
APACHE II score	18.1 ± 7.5	11.7 ± 3.9	<.0001
Sex, male (%)	60.7	60.7	1.0000
Body weight, kg	53.2 ± 11.7	57.9 ± 12.6	0.0561
Height, cm	159.1 ± 1.8	159.8 ± 0.43	0.7302
Age, years	57.3 ± 3.0	62.1 ± 0.8	0.127
Medical admission, %	42.86	7.4	<.0001
pH	7.41 ± 0.076	7.42 ± 0.037	0.3978
PaCO_2_, mmHg	38.3 ± 5.2	40.5 ± 5.3	0.0331
Sodium, mmol/L	131.1 ± 4.7	133.0 ± 4.0	0.0166
Potassium, mmol/L	3.9 ± 0.6	3.8 ± 0.38	0.0298
Calcium, mmol/L	1.1 ± 0.098	1.1 ± 0.064	0.0001
Albumin, g/dL	2.75 ± 0.65	2.83 ± 0.51	0.41
Chloride, mmol/L	99.8 ± 5.7	102.9 ± 4.2	0.0002
Bicarbonate, mmol/L	24.4 ± 4.1	26.0 ± 2.8	0.0059
Lactate, mmol/L	2.9 ± 5.1	1.2 ± 0.9	<.0001
SBE, mmol/L	0.33 ± 4.6	1.9 ± 3.7	0.0043
SIDa, mmol/L	33.4 ± 4.5	33.7 ± 3.9	0.6904
AG, mmol/L	10.7 ± 6.5	7.9 ± 3.3	<.0001
Hematocrit, %	30.2 ± 5.4	32.6 ± 4.8	0.0109
Glucose, mg/dL	159.8 ± 66.6	149.0 ± 37.6	0.1634

APACHE II score: Acute Physiology and Chronic Health Evaluation II score; SBE: standard base excess; SIDa: apparent strong ion difference; AG: anion gap.

**Table 5:** Logistic regression model for survival.

Variables	Coefficient	Odds ratio	*P* value
		(95% confidence interval)	
APACHE II score	0.1821	1.200 (1.081–1.331)	0.000619
Medical admission	0.9544	2.597 (1.437–4.693)	0.001573
PaCO_2_	0.01246	1.012 (0.890–1.151)	0.8497
Sodium	−0.1104	0.8954 (0.7380–1.086)	0.2629
Potassium	0.7253	2.065 (0.6817–6.257)	0.1996
Chloride	0.05399	1.055 (0.8944–1.246)	0.5227
Calcium	−6.124	0.00219 (2.170 × 10^−6^–2.209)	0.08267
Bicarbonate	−0.03445	0.9661 (0.7341–1.272)	0.8058
Lactate	0.5559	1.743 (1.138–2.671)	0.01069
Hematocrit	−0.00603	0.9940 (0.8931–1.106)	0.9120

**Table 6:** Comparison of variables between medical and surgical admissions.

Variables	Medical (*n* = 46)	Surgical (*n* = 442)	*P* value
Age, years	59.4 ± 15.6	62.1 ± 16.2	0.2827
Sex, male (%)	43.5%	38.9%	0.6346
pH	7.43 ± 0.06	7.42 ± 0.03	0.226
PaCO_2_, mmHg	40.8 ± 7.4	40.4 ± 5.0	0.5504
Sodium, mmol/L	133.0 ± 7.1	132.9 ± 3.6	0.8201
Potassium, mmol/L	3.88 ± 0.58	3.75 ± 0.377	0.041
Chloride, mmol/L	98.8 ± 6.4	103.1 ± 3.9	<.0001
Calcium, mmol/L	1.12 ± 0.10	1.13 ± 0.06	0.3973
Bicarbonate, mmol/L	26.7 ± 4.8	25.8 ± 2.7	0.0354
Lactate, mmol/L	1.9 ± 4.0	1.3 ± 1.0	0.0073
Glucose, mg/dL	150.7 ± 58.9	149.5 ± 37.4	0.8572
Hematocrit, %	31.6 ± 6.5	32.6 ± 4.7	0.1903
Albumin, g/dL	2.85 ± 0.46	2.82 ± 0.52	0.6877
Total bilirubin, mg/dL	2.1 ± 2.9	1.4 ± 1.7	0.0138

## Abbreviations

SID: Strong ion difference
ICU: Intensive care unit
APACHE II score: Acute Physiology and Chronic Health Evaluation II score
SIDa: Apparent strong ion difference
SIDe: Effective strong ion difference
SIG: Strong ion gap
SBE: Standard base excess
AG: Anion gap.
